# Supplementary material for: Post-Harvest LED Light Irradiation Affects Firmness, Bioactive Substances, and Amino Acid Compositions in Chili Pepper (Capsicum annum L.)
Source: Foods. 2022 Sep 5;11(17):2712. doi: 10.3390/foods11172712 (PMC9455159; doi:10.3390/foods11172712)
Supplement: Supplementary file 1 [file foods-11-02712-s001.zip › Table S1_ 20220902.pdf]

Table S1. Effect of light quality on the amino acid composition and contents (g kg^{-1}) in fruits of three pepper cultivars.

	Hangjiao-2				Xinxiang-2				P1622			
	Dark	White	Red	Blue	Dark	White	Red	Blue	Dark	White	Red	Blue
Asp^{BD}	2.6 ±0.2b	2.1 ±0.1c	4.5 ±0.3a	4.4 ±0.1a	3.3 ±0.2a	2.7 ±0.1b	2.4 ±0.1c	3.4 ±0.1a	3.4 ±0.2a	2.6 ±0.1b	2.6 ±0.1b	3.2 ±0.2a
Thr^{A}	1.9 ±0.1d	2.1 ±0.1c	3.0 ±0.2a	2.8 ±0.1b	1.9 ±0.1ab	1.9 ±0.1b	1.9 ±0.1b	2.0 ±0.1a	1.9 ±0.1a	1.6 ±0.1b	1.7 ±0.1ab	1.6 ±0.2b
Ser^{BE}	2.3 ±0.1d	2.4 ±0.1c	3.3 ±0.2a	3.1 ±0.2b	2.2 ±0.1a	1.9 ±0.1b	1.8 ±0.1b	2.2 ±0.2a	2.2 ±0.1a	1.9 ±0.1b	1.9 ±0.1b	1.9 ±0.1b
Glu^{BD}	4.5 ±0.3b	4.7 ±0.2b	6.6 ±0.5a	6.4 ±0.4a	4.7 ±0.3a	3.9 ±0.2b	3.6 ±0.2c	4.8 ±0.4a	4.6 ±0.2a	3.8 ±0.2c	4.2 ±0.2b	4.0 ±0.2bc
Pro^{BE}	2.0 ±0.1c	2.5 ±0.1b	3.1 ±0.3a	3.0 ±0.2a	2.1 ±0.1b	2.3 ±0.1b	2.6 ±0.2a	2.3 ±0.1b	2.1 ±0.1a	1.9 ±0.1b	1.9 ±0.1b	1.9 ±0.1b
Gly^{BE}	2.2 ±0.1c	2.7 ±0.2b	3.4 ±0.2a	3.2 ±0.3a	2.3 ±0.1a	2.3 ±0.2a	2.4 ±0.2a	2.4 ±0.1a	2.2 ±0.1a	2.0 ±0.1b	2.0 ±0.1b	1.9 ±0.2b
Ala^{BE}	2.5 ±0.2d	2.9 ±0.2c	3.8 ±0.3a	3.6 ±0.2b	2.5 ±0.1ab	2.3 ±0.1b	2.3 ±0.1b	2.6 ±0.2a	2.5 ±0.1a	2.1 ±0.1b	2.3 ±0.2b	2.1 ±0.1b
Val^{A}	2.0 ±0.1c	2.4 ±0.1b	2.9 ±0.2a	2.8 ±0.2a	2.0 ±0.1a	1.9 ±0.1a	1.8 ±0.1a	2.0 ±0.1a	1.8 ±0.1a	1.6 ±0.1b	1.7 ±0.1b	1.7 ±0.1b
Met^{A}	0.50 ±0.03d	0.54 ±0.04c	0.85 ±0.06a	0.62 ±0.04b	0.51 ±0.02b	0.48 ±0.03c	0.49 ±0.02c	0.55 ±0.02a	0.53 ±0.02a	0.41 ±0.03b	0.38 ±0.02bc	0.36 ±0.02c
Ile^{A}	1.5 ±0.1c	1.8 ±0.1b	2.3 ±0.2a	2.2 ±0.1a	1.5 ±0.1a	1.6 ±0.1a	1.5 ±0.1a	1.6 ±0.2a	1.4 ±0.1a	1.3 ±0.1a	1.3 ±0.1a	1.4 ±0.1a
Leu^{A}	3.7 ±0.2c	4.6 ±0.4b	5.7 ±0.3a	5.5 ±0.4a	3.8 ±0.3a	3.8 ±0.3a	3.8 ±0.2a	4.0 ±0.3a	3.6 ±0.2a	3.3 ±0.1b	3.3 ±0.1b	3.2 ±0.1b
Tyr^{BF}	1.7 ±0.1c	2.1 ±0.1b	2.3 ±0.2a	2.4 ±0.1a	1.7 ±0.1b	1.7 ±0.1a	1.5 ±0.1c	1.7 ±0.1a	1.5 ±0.1a	1.4 ±0.1a	1.5 ±0.1a	1.4 ±0.1a
Phe^{BF}	2.3 ±0.1c	2.9 ±0.2b	3.4 ±0.2a	3.3 ±0.2a	2.3 ±0.2a	2.4 ±0.1a	2.4 ±0.2a	2.5 ±0.2a	2.2 ±0.1a	2.0 ±0.1b	2.1 ±0.1b	2.0 ±0.1b
His^{BC}	0.43 ±0.03c	0.28 ±0.03d	1.3 ±0.1a	1.0 ±0.1b	0.72 ±0.04b	0.75a ±0.03b	0.79 ±0.03a	0.67 ±0.03c	0.99 ±0.05a	0.53 ±0.04c	0.44 ±0.04d	0.83 ±0.06b
Lys^{A}	2.8 ±0.2c	3.4 ±0.2b	4.2 ±0.3a	4.1 ±0.3a	2.8 ±0.2b	2.7 ±0.2b	2.4 ±0.1c	2.9 ±0.1a	2.6 ±0.1a	2.4 ±0.1b	2.5 ±0.1b	2.4 ±0.1b
Arg^{BC}	2.7 ±0.2c	3.0 ±0.2b	3.4 ±0.2a	3.5 ±0.3a	2.6 ±0.1b	2.2 ±0.2c	1.9 ±0.1c	2.9 ±0.2a	2.8 ±0.2a	2.5 ±0.1b	2.3 ±0.1b	2.7 ±0.1a
Cys^{B}	0.14 ±0.01b	0.14 ±0.01b	0.21 ±0.01a	0.20 ±0.01a	0.16 ±0.01b	0.17 ±0.01ab	0.15 ±0.01b	0.18 ±0.01a	0.19 ±0.02a	0.15 ±0.01c	0.14 ±0.01d	0.16 ±0.01b

Note: Amino acids are represented by the 3-letter abbreviation code. A, Essential amino acid; B, Non-essential amino acid; C, Children essential amino acid; D, Monosodium glutamate-like amino acid; E, Sweet amino acid; F, Aromatic amino acid. The results are shown as the mean ± SE of triplicate samples. Means denoted by the same letter did not differ significantly at $p < 0.05$ according to Tukey's test.
