# Supplementary figures and images for: Post-Harvest LED Light Irradiation Affects Firmness, Bioactive Substances, and Amino Acid Compositions in Chili Pepper (Capsicum annum L.)
Source: Foods. 2022 Sep 5;11(17):2712. doi: 10.3390/foods11172712 (PMC9455159; doi:10.3390/foods11172712)

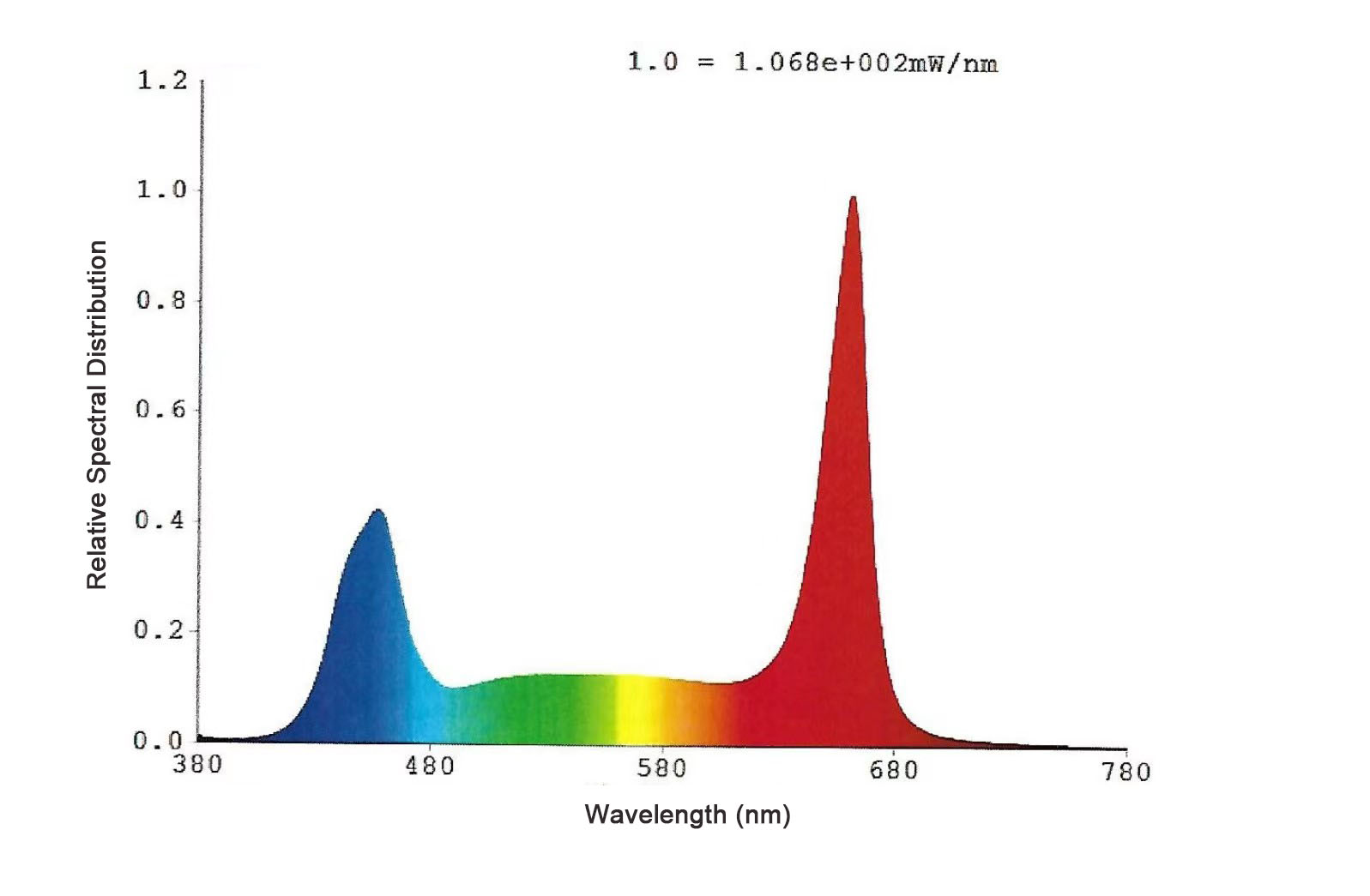

Supplement: Supplementary file 1 [file foods-11-02712-s001.zip › Figure S1 The spectrum of the LED white light we used in this study.jpg]
